# Health Literacy, Social Support and Quality of Life in Chinese Health Examination Population

**DOI:** 10.3390/healthcare14142122

**Published:** 2026-07-15

**Authors:** Yuanyuan Song, Wei Xie, Bo Zhang, Xirong Hu, Jing Wang, Qiaoling Li

**Affiliations:** 1 Faculty of Nursing, Health Science Center, Xi’an Jiaotong University, Xi’an 710061, China; 2 Department of Nursing, The First Affiliated Hospital of Xi’an Jiaotong University, Xi’an 710061, China; 3 Healthcare Management Center, Xi’an No.1 Hospital, Xi’an 710001, China

**Keywords:** physical examination, health literacy, social support, quality of life

## Abstract

**Background**: Health literacy has become an important determinant of population health. Understanding health literacy levels among physical examination populations in China and examining their associations with social support and quality of life can provide evidence to guide preventive health strategies. **Methods**: A cross-sectional survey was conducted from May 2023 to May 2024 among 416 individuals undergoing routine physical examinations at a tertiary hospital in Xi’an, China. Participants completed a socio-demographic characteristics questionnaire, the Health Literacy Surveillance Rapid Assessment Questionnaire, the Social Support Rating Scale and the Euro-Qol five-dimension questionnaire. Descriptive statistics, correlation analyses, and multiple linear regression models were used to examine health literacy status, associated factors, and the former’s relationships with social support and quality of life. **Results**: Health literacy in the physical examination population was higher than the national average, and the proficiency rate was 52.4%. The findings indicate a significant and positive association between social support and health literacy (r = 0.398, *p* < 0.01); health literacy was found to be significantly and positively associated with quality of life (r = 0.216, *p* < 0.01). According to the multiple linear regression analysis, factors correlated with health literacy included age, gender, place of residence, marital status, educational level, monthly household income, payment method for physical examinations, frequency of physical examinations, and social support. **Conclusions**: Enhancing the health education competencies of medical personnel to enable effective dissemination of health information during physical examinations, promoting family involvement in health management to cultivate collective health awareness and practices, and improving the medical insurance system to encourage regular physical examinations are effective strategies for strengthening public health literacy.

## 1. Introduction

The relentless rise in non-communicable disease incidence and escalating healthcare costs are exerting unprecedented pressure on health systems worldwide [1], underscoring the urgent need to develop effective preventive strategies. In this context, the improvement of personal health management capabilities has become a key factor in enhancing the effectiveness of public health and in achieving the sustainable development of medical services. Health screening serves as a cornerstone of preventive healthcare, playing a fundamental role in early disease detection and the advancement of public health [2]. A cross-sectional study in Japan showed that health literacy is significantly associated with physical examination attendance [3], suggesting that physical examination may provide an “educational opportunity” for health literacy intervention and play an important role in the prevention and control of non-communicable diseases. However, the effectiveness of screening programs depends not only on clinical services but is also closely linked to health literacy, which refers to individuals’ knowledge and competencies related to maintaining and promoting health and can be reflected in basic health knowledge and concepts, healthy lifestyle and behaviors, and health skills [4]. Research demonstrates that low health literacy is associated with adverse outcomes, including medication errors, reduced utilization of preventive services, poor chronic disease management, and exacerbated health disparities [5,6,7]. Adequate health literacy enables individuals to acquire health knowledge, manage their health and develop healthier lifestyles [8,9].

Although the importance of health literacy is increasingly recognized, existing research mainly focuses on clinical patients or the general public rather than those seeking preventive health care [10], which may also include individuals with underlying risk factors or those in the early stages of disease. This group is at a critical transition period, and the effective communication of screening results and subsequent health recommendations will significantly affect their future health trajectory [11,12]. Therefore, identifying factors associated with health literacy in this group may help health-care professionals develop more targeted education and communication strategies during the examination process.

Research demonstrates that social support plays a significant role in developing and maintaining health literacy [13]. Social support is the process by which individuals feel loved, valued and needed. High levels of social support can protect individuals from the negative effects of stressful events and help them better cope with environmental challenges [14]. This support system encompasses not only emotional backing from family, friends, and communities, but also practical assistance and informational guidance, all of which enhance individuals capacity to tackle health challenges [14]. Improving social support has been associated with healthier behaviors, better health management, and improved outcomes in patients with diseases such as coronary heart disease [15] and hypertension [16]. For individuals undergoing routine physical examinations, the significance of health literacy extends beyond the acquisition of health knowledge. Physical examinations generate health information that needs to be understood, interpreted, and translated into daily health management behaviors. These abilities have been shown to contribute to better quality of life [17]. The factors associated with health literacy are multidimensional, including socioeconomic status, education level, gender, cultural background, previous health experience, etc., [8,18].

This study investigates health literacy levels among individuals undergoing physical examinations, analyzing its interrelationships with social support and quality of life. Accordingly, this study aimed to describe the level of health literacy among individuals undergoing routine physical examinations and to examine demographic, socioeconomic, health examination-related, and social support factors associated with health literacy. In addition, this study explored the associations of health literacy and social support with quality of life. We hypothesized that higher social support would be positively associated with health literacy, and that higher health literacy would be positively associated with better quality of life. By identifying factors associated with health literacy and examining its associations with social support and quality of life, this study may provide evidence to inform health education and communication strategies in health examination settings.

## 2. Methods

### 2.1. Study Design and Participants

#### 2.1.1. Sampling Method

A non-probability convenience sampling method was used. From May 2023 to May 2024, individuals undergoing physical examinations at a tertiary hospital in Xi’an, China, who met the inclusion and exclusion criteria were invited to participate in this study.

#### 2.1.2. Inclusion and Exclusion Criteria

The inclusion criteria of this study were: a. participants aged 18 years or older, with normal comprehension and the ability to independently answer questions or complete questionnaires; b. individuals who voluntarily participated in the study. The exclusion criteria were: a. individuals undergoing occupational health examinations; b. those who lacking self-control or with cognitive or sensory impairments; c. individuals unable to cooperate or communicate effectively; d. those currently experiencing illness or receiving medical treatment.

#### 2.1.3. Sample Size

The sample size was estimated using the formula for a single population proportion: *n* = *Z*^2^*p*(1 − *p*)/*d*^2^, Z is the standard normal value for the selected confidence level, *p* is the anticipated proportion of individuals with adequate health literacy, and d is the allowable margin of error. In the absence of a reliable estimate of the adequate health literacy rate among a comparable health examination population, *p* was conservatively set at 0.50. With a 95% confidence level (*Z* = 1.96) and a margin of error of 5% (*d* = 0.05), the minimum required sample size was 384 participants. After allowing for an anticipated 10% rate of invalid questionnaires, the target sample size was 427 participants. A total of 436 questionnaires were collected.

### 2.2. Measures

#### 2.2.1. Socio-Demographic Characteristics Questionnaire

The study used a self-administered questionnaire that included general information such as age, gender, ethnicity, marital status, place of residence, educational level, household income, method of payment for health examination fees, frequency of health examinations, and family medical history.

#### 2.2.2. Health Literacy Surveillance Rapid Assessment Questionnaire

In 2017, based on the National Health Literacy Monitoring Survey questionnaire, Chinese scholar Du Xiuben [19] developed a shortened version of the National Health Literacy Monitoring Rapid Assessment Questionnaire. The 19-item scale encompasses three domains: health knowledge and concepts (7 items), healthy lifestyle and behaviors (6 items), and basic health skills (6 items). A score of one point is awarded for each question, with a total score of 19 points. A score of 16 (out of 19) indicates adequate health literacy. The Cronbach’s α coefficient was 0.82. It can be applied to the rapid assessment of health literacy in different populations.

#### 2.2.3. Social Support Rating Scale

Developed by Chinese scholar Xiao [20] in 1986, this scale consists of 10 items, including objective support including three items, subjective support with four items, and social support utilization with three items.

The scoring principle is as follows: Items 1–4 and 8–10 are scored positively, with scores ranging from 1 to 4 points per item. For item 5, scores range from 1 to 4 corresponding to responses from “none” to “full support”. For items 6 and 7, if “no sources” is selected, the score is 0, and if “the following sources” is chosen, scores are cumulatively calculated based on the number of selections. The total score of this scale is 66 points and the level of social support is evaluated as follows: low level (0–22 points), medium level (23–44 points), and high level (≥45 points). The Cronbach’s α coefficient of the Social Support Rating Scale was 0.91 in this study.

#### 2.2.4. Euro-Qol Five-Dimension Questionnaire

The Euro-Qol five-dimension questionnaire (EQ-5D) [21] is a standardized measure of health-related quality of life (HRQoL) developed by the European Society for Quality of Life (EuroQol Group) to assess and describe health status. EQ-5D measures an individual’s quality of life through five dimensions, including mobility, self-care, daily activities, pain or discomfort, and anxiety or depression. Each dimension comprises three levels of responses: level 1 indicates the absence of problems, level 2 indicates the presence of moderate problems, and level 3 indicates the presence of severe problems. The Chinese version of the EQ-5D-3L was used in this study. Although studies on EQ-5D utility value conversion have been conducted in China, no universally accepted conversion standard has been established. Therefore, considering the cultural and lifestyle similarities between China and Japan, the Japanese time trade-off (TTO) value set was applied to calculate health utility values [22]. The utility score was calculated as 1.00 minus the corresponding values for each dimension and the constant term, as shown in Table 1. Scores ranged from −0.11 to 1.00, with higher scores indicating better health-related quality of life.

### 2.3. Data Collection

The data collection process was conducted via Wenjuanxing (WJX), a Chinese online questionnaire platform (www.wjx.cn). WJX codes were printed and posted at the reception desk of the health examination center. Upon obtaining informed consent from the participants, they were requested to scan the QR code to complete the questionnaire. The entire survey process was anonymous, and quality control was ensured through the application of the WJX platform, which allowed questionnaires to be submitted only after all items had been completed.

### 2.4. Statistical Analysis

Data was analyzed using SPSS version 22.0. Descriptive statistics, such as “mean ± standard deviation”, were used to describe continuous data. The Categorical data were summarized in terms of frequency and percentage. One-way analysis of variance (ANOVA) test was used to compare the means of three or more groups while the independent samples *t*-test, one of the parametric statistical methods, was used to compare the means of two independent groups. The normality of continuous variables was assessed using the Shapiro–Wilk test, and Pearson or Spearman correlation coefficients were selected according to the distribution of the data to examine associations between continuous variables. Multiple linear regression analysis was applied to investigate the factors associated with health literacy among health examination participants. The level of statistical significance was set at *p* < 0.05.

### 2.5. Ethical Consideration

This study was approved by the First Affiliated Hospital of Xi’an Jiaotong University, Biomedical Ethics Committee with the Approval Code: 2022-1615 on 6 January 2022. The staff explained the purpose, the content, the benefits and potential risks, and participants’ rights to all individuals undergoing physical examinations who met the inclusion criteria on site. In this study, participants were asked to read the informed consent form and tick the agreement box before starting the questionnaire. All participants were assured of anonymity and could withdraw at any time.

## 3. Results

### 3.1. Socio-Demographic Characteristics

A total of 436 questionnaires were distributed in this survey, of which 416 were valid, with an effective rate of 95.4%. Table 2 presents the socio-demographic information of the participants. In this study, 44.5% of the participants were male. The age range of the participants undergoing physical examinations was 19 to 79 years, and the mean age was 42.69 ± 14.99 years. The majority of the participants was married (73.1%) and Han Chinese (89.7%). Up to 72.1% of participants lived in cities, and 65.1% had a junior college or bachelor’s degree or above. A small proportion (10.6%) reported having religious beliefs, and approximately one-third of the participants were retired or unemployed. Regarding household income, 24.8% had a monthly per capita household income of less than 3000 yuan, while 43.5% reported a monthly per capita household income exceeding 5000 yuan. In terms of the payment method, 37.7% had medical insurance, 22.1% had their medical examination expenses covered by their employers, and 40.1% paid out-of-pocket. Regarding the frequency of health check-ups, 40% underwent a physical examination annually, 40% every 2–3 years, and 20% less frequently (every four years or more). Additionally, 4.8% of participants reported a family history of genetic disorders.

### 3.2. Health Literacy, Social Support and Quality of Life in Study Participants

Table 3 shows the scores of Health literacy, Social support and Quality of life in the study participants. Overall, 52.4% of participants had an adequate level of Health literacy.

The Shapiro–Wilk tests indicated departures from normality for several continuous variables. However, visual inspection of histograms and Q–Q plots suggested only mild deviations from normality, without substantial skewness or influential extreme outliers. Considering the relatively large sample size (*n* = 416). and the exploratory purpose of the correlation analysis, Pearson’s correlation coefficients were used to quantify linear associations among continuous variables.

The Pearson correlation analysis results indicate a positive correlation between health literacy and social support (r = 0.398, *p* < 0.01). Health literacy was positively linked with quality of life (r = 0.216, *p* < 0.01). Moreover, there was a positive correlation between social support and quality of life (r = 0.179, *p* < 0.01). The correlations between the dimensions are shown in Table 4.

### 3.3. Multiple Linear Regression Analysis of Health Literacy

The results from the multiple linear regression analyses are reported in Table 5. Based on previous literature [8,13,18] and the objectives of this study, sociodemographic characteristics, health related factors, and total social support score were included as independent variables in the multiple linear regression model. The results showed that high health literacy was associated with the following variables: gender, age, marital status, education level, annual family income, payment method and frequency of physical examination and social support were the influencing factors of health literacy. Variables in the model explained 40.7% of the variance of health literacy (F = 13.552, *p* < 0.001). The maximum VIF < 10, indicating that multicollinearity was not present in the model.

## 4. Discussion

In this study, we explored the level of health literacy among individuals undergoing health examinations and examined the associations among the social support, health literacy and quality of life and the factors associated with health literacy. These findings provide an overview of the relationships observed among individuals undergoing health examinations. Specifically, there were three key findings. First, the health literacy of the participants in this study was found to be at a relatively high level. Second, significant positive correlations were observed between health literacy, social support, and quality of life. Third, age, gender, marital status, educational level, monthly household income, payment method for physical examinations, frequency of physical examinations, and social support were associated with health literacy.

The participants in this study were individuals undergoing health examinations, whose health literacy rate was 52.40%, which was higher than the national average of 33.69% [23]. In the study, health literacy was assessed as a multidimensional construct encompassing not only health knowledge and competencies related to obtaining, understanding, and using health information, but also healthy lifestyle practices and health-related behaviors. Therefore, the observed health literacy level should be interpreted as reflecting both health information-related competencies and engagement in recommended health-related behaviors. This difference may be partly related to the characteristics of individuals who undergo health examinations, such as greater attention to health, more frequent contact with health services, or better access to health information [24]. These may be possible reasons why individuals undergoing health examinations had higher health literacy levels than the national average.

In this study, the social support was positively correlated with health literacy among the participants (*p* < 0.05). This finding is consistent with the research results of Mo, Zhang and other scholars [15,25]. In the multivariable analysis, social support was also independently associated with health literacy. Previous studies have suggested that social support is associated with access to health care, health information, and healthy behaviors [26]. Among individuals undergoing health examinations, social support may be associated with greater access to health information, health skills, and understanding of health-related knowledge and practices. Social support also has been reported to be closely associated with an individual’s health status [27]. Therefore, assessing an individual’s social support status may help identify those who could benefit from additional health information and support resources. Furthermore, a positive correlation was found between health literacy and quality of life (*p* < 0.05), suggesting that higher health literacy levels in the health check-up population are associated with better quality of life. This finding is consistent with previous studies that have also reported a positive correlation between health literacy and quality of life [28]. Consistent with several studies, our study also found a statistically significant positive association between social support and quality of life [15,29]. Overall, these findings suggest that health literacy, social support, and quality of life may be interrelated among individuals undergoing physical examinations. Interventions that integrate health education with appropriate social support may be worthy of further evaluation as potential approaches to support health-related outcomes and quality of life in this population.

In this study, age was found to be associated with health literacy, with older individuals exhibiting higher levels of health literacy. This finding contrasts with the prevailing literature, which often reports that advanced age is associated with poorer physical health and comprehension abilities, potentially impairing the capacity to access, understand, evaluate, and apply health information, thereby reducing health literacy [30,31]. The discrepancies between our findings and those of previous studies may be attributed to variations in sample characteristics. With the increase in age, the prevalence of chronic diseases will also increase [32], and people may consequently have more opportunities to pay attention to and accumulate knowledge related to disease prevention and treatment. The elderly may also be exposed to relevant health information through cancer screening items included in physical examinations, such as colonoscopy and PSA test [33,34]. And older, healthier people are more likely to proactively undergo annual physicals or screenings than younger people who seek medical care only because they are sick [11]. These may be the reasons why older people in the physical examination group had higher health literacy levels. On the contrary, young people have better physical fitness but pay less attention to health literacy [35]. Meanwhile, they face greater life and work pressures [36], forcing them to devote less time to their own health.

It is worth noting that this study found that the health literacy of married people was lower, which contradicted the conclusion of Sulinskaite that the family-oriented social support level of married groups was higher [37]. A reasonable explanation is that unmarried or divorced people, without a partner, may need to take more personal responsibility for their health management, and thus be more active in accessing health information and improving health literacy. In marriage, the task of health management is often divided into specialized roles and delegated to a fixed partner. In this way, the other party may become a passive recipient of care, resulting in the deterioration of their ability to query personal health information, critical evaluation ability and self-management efficacy in the medical system. Women often take on more responsibility for family care [38]. It is usually the woman who makes family appointments, remembers medication, studies the condition and contacts the medical provider. This may also be the reason why women have higher health literacy. Therefore, when developing intervention strategies, it is crucial to strengthen family support networks while empowering individuals. Implementing “co-participation by spouses” in healthcare practices is important, such as actively encouraging patients to bring their partners during appointments and follow-ups. Additionally, male-oriented health information channels and programs could be developed, for example by integrating health content into sports media, automotive forums, or financial applications, and health screening stations could be established in gyms and fitness centers. At the same time, men’s participation in family health management should be encouraged by promoting the concept of “shared wellness” as an essential aspect of marital care and family well-being, rather than viewing health management as solely the responsibility of women.

Household income was associated with health literacy levels. One study that specifically examined the relationship between socioeconomic status and health literacy showed similar results to the present work [8]. Higher income may be associated with better access to health resources, preventive services, and health information, whereas individuals with lower incomes may face greater financial and life pressures when engaging with preventive health services. Improving population health literacy is recognized as an effective way to reduce health disparities among social strata [25]. However, there was no significant difference between the high-income and middle-income groups, which should be interpreted cautiously because the present study was not designed to test a threshold effect. Therefore, in the development of health promotion strategies, greater attention should be given to improving health education and the accessibility of health resources among low-income populations. Potential approaches include providing low-cost or free physical examination services, strengthening health communication channels, and developing educational materials that are culturally appropriate and tailored to the cognitive levels of target groups, which may help improve access to and use of health information among disadvantaged populations. Different payment methods for physical examinations were also related to health literacy levels. Individuals who paid for physical examinations with medical insurance had higher levels of health literacy than those who paid for physical examinations by themselves or their employers. A study conducted in the United States revealed that health insurance is associated with increased likelihood of undergoing regular physical examinations [11]. Increasing the health examination items in medical insurance may reduce financial barriers to participation in health examinations and may create more opportunities to access preventive health services.

In this study, individuals who underwent regular annual health check-ups showed lower levels of health literacy compared to those with less frequent check-ups. In this study, individuals who underwent regular annual health check-ups showed lower levels of health literacy compared to those with less frequent check-ups. This association should be interpreted cautiously, as the reasons for attending health examinations at different frequencies were not assessed in the present study. It does not indicate that more frequent health examinations reduce health literacy.

## 5. Limitation

The present study has several limitations. First, the cross-sectional design precludes the identification of causal associations between the main variables. Second, owing to the limited of the study period, the sample size is relatively modest and was recruited from a single hospital in Northwest China. This may have afected the representativeness of the sample and the generalizability of the findings. In addition, all participants were individuals undergoing health examinations; therefore, the findings may not be generalizable to individuals who do not participate in health examinations, and direct comparisons between these groups were not possible. Third, due to the limitations of objective constraints and the relatively narrow scope of evaluation variables, some potential psychosocial and behavioral factors related to health literacy were not included in the analysis, which may restrict the comprehensiveness of the findings. Moreover, the use of self-reported questionnaires may have introduced reporting bias. Finally, the conclusion that higher education level is associated with a higher health literacy level was not verified in this study, and further research on the reasons is needed.

Despite these limitations, this study also has several notable contributions. By focusing on individuals undergoing routine physical examinations, the study captured health literacy and its influencing factors at the frontline of disease prevention, a context that has received limited attention in previous research. Moreover, the findings highlight the prominent role of social support in shaping health literacy, underscoring the importance of social environments in health promotion. The integrated examination of health literacy, social support, and quality of life further provides empirical evidence for developing health education strategies within preventive health service settings.

## 6. Conclusions

In our study, health literacy was associated with a variety of factors and social support was found to have a particularly significant association with health literacy levels. These findings suggest that efforts may focus on empowering individuals through community-based health education programs, establishing multi-level support networks that connect families, peers, and healthcare providers, and enhancing people’s ability to identify and use reliable health information sources. In addition, promoting family involvement in health communication and fostering collaborations between healthcare institutions, community organizations, and policymakers may help create a supportive environment for individuals to acquire, understand, and apply health information. Social media platforms may also be considered as channels for disseminating health knowledge and improving access to health information, particularly among socioeconomically disadvantaged groups. Moreover, as an important setting for preventive healthcare, physical examination services may be used by healthcare providers to disseminate health knowledge, advocate healthy lifestyles, and improve population health literacy, thereby potentially contributing to improved quality of life.

## Figures and Tables

**Table 1:** Japanese time trade-off (TTO) value set for the EQ-5D-3L.

Dimension	No Problem	Moderate Problems	Serious Problems	Constant
Action	0.000	0.075	0.418	
Self-care	0.000	0.054	0.102	
Usual activities	0.000	0.044	0.133	
Pain/discomfort	0.000	0.080	0.194	
Anxiety/depression	0.000	0.063	0.112	
				0.152

**Table 2:** Participant’s characteristics and their HL (*n* = 416).

Variables		Total Sample *n*(%)/$\bar{x}\pm s$ (Range)	HL ^a^ $\bar{x}\pm s$	t/F/r	*p*
Gender					
	Male	185 (44.5)	13.39 ± 5.17	−1.401 ^c^	0.162
	Female	231 (55.5)	14.09 ± 5.00		
Age		42.69 ± 14.99 (19~79)		−0.950 ^e^	0.054
Ethnicity					
	Han	373 (89.7)	13.71 ± 5.13	−0.776 ^c^	0.438
	Minority	43 (10.3)	14.35 ± 4.70		
Marital status					
	Unmarried	94 (22.6)	14.44 ± 4.35	1.767 ^d^	0.172
	Married	304 (73.1)	13.67 ± 5.18		
	Divorced/separated/widowed	18 (4.3)	12.17 ± 6.56		
Residence					
	City	300 (72.1)	14.24 ± 4.56	5.544 ^d^	0.004 **
	Country	60 (14.4)	13.20 ± 5.97		
	Rural	56 (13.5)	11.91 ± 6.14		
Educational level					
	Less than senior high school education	89 (72.1)	12.26 ± 6.49	12.538 ^d^	<0.001 ***
	Senior high school or technical secondary school education	56 (21.4)	11.91 ± 5.66		
	College degree or above	271 (13.5)	14.66 ± 4.15		
Religious					
	Yes	44 (10.6)	12.84 ± 5.08	1.296 ^c^	0.196
	No	372 (89.4)	13.89 ± 5.08		
Employment status					
	Employed	276 (66.3)	14.32 ± 4.73	5.138 ^d^	0.006 **
	Retired	73 (17.5)	12.34 ± 5.79		
	Unemployed	67 (16.1)	13.12 ± 5.36		
Per capita monthly household income					
	≤3000 CNY ^b^	103 (24.8)	12.09 ± 6.03	17.370 ^d^	<0.001 ***
	3001~5000 CNY ^b^	132 (31.7)	12.95 ± 5.10		
	≥5001 CNY ^b^	181 (43.5)	15.35 ± 3.92		
Payment method of physical examination fee					
	At one’s own expense	167 (40.1)	12.05 ± 5.16	21.560 ^d^	<0.001 ***
	The work unit pays	92 (22.1)	13.84 ± 4.59		
	Health insurance card	157 (37.7)	15.59 ± 4.65		
Frequency of physical examination					
	Annually	165 (39.7)	12.84 ± 5.22	4.828 ^d^	0.008 **
	Every 2–3 years	167 (40.1)	14.29 ± 4.44		
	Every 4 years or more	84 (20.2)	14.60 ± 5.74		
Family genetic history					
	Yes	20 (4.8)	13.89 ± 5.03	1.972 ^c^	0.049 *
	No	396 (95.2)	11.60 ± 5.77		

^a^: HL: Health literacy. ^b^: CNY Chinese yuan (¥). ^c^: Independent-samples *t*-test statistic (t value); ^d^: one-way analysis of variance statistic (F value); ^e^: Pearson correlation coefficient (r value). *: *p* < 0.05, **: *p* < 0.01, ***: *p* < 0.001.

**Table 3:** Health literacy, Social support and Quality of life of participants (*n* = 416).

Variables	Dimension Score/$\bar{x}\pm s$
Total health literacy score ^a^	13.38 ± 5.08
Basic knowledge and ideas	5.37 ± 1.90
Healthy lifestyle and behavior	4.57 ± 1.83
Basic skill	3.83 ± 1.86
Total social support score	23.06 ± 5.51
Objective support	9.97 ± 3.51
Subjective support	7.11 ± 2.01
Social support utilization degree	7.11 ± 2.01
Total quality of life score	0.82 ± 0.07
Action	1.03 ± 0.18
Self-care	1.03 ± 0.19
Usual activities	1.04 ± 0.21
Pain/discomfort	1.17 ± 0.38
Anxiety/depression	1.21 ± 0.48

^a^: Health literacy Score rate is 52.40%.

**Table 4:** Correlation between health literacy, social support, and quality of life of participants.

	Basic Knowledge and Ideas	Healthy Lifestyle and Behavior	Basic Skill	Total Social Support Score	Objective Support	Subjective Support	Social Support Utilization Degree	Total Quality of Life Score
Total health literacy score	0.924 **	0.922 **	0.880 **	0.398 **	0.489 **	0.180 **	0.275 **	0.216 **
Basic knowledge and ideas	1	0.816 **	0.700 **	0.404 **	0.432 **	0.223 **	0.282 **	0.253 **
Healthy lifestyle and behavior		1	0.701 **	0.347 **	0.457 **	0.136 **	0.243 **	0.171 **
Basic skill			1	0.333 **	0.444 **	0.130 **	0.224 **	0.165 **
Total social support score				1	0.635 **	0.866 **	0.594 **	0.179 **
objective support					1	0.230 **	0.209 **	0.217 **
Subjective support						1	0.386 **	0.109 *
Social support utilization degree							1	0.053

*: *p* < 0.05, **: *p* < 0.01.

**Table 5:** A model of multiple linear regression with HL as a dependent variable (*n* = 416).

Variables	B	SE	β	*t*	*p*	VIF
(constant)	−2.32	1.599		−1.451	0.148	
Age	0.061	0.029	0.18	2.135	0.033 *	4.710
Ethnicity						
Han (Ref.)						
Minority	1.550	1.172	0.055	1.323	0.187	1.155
Marital status						
Unmarried (Ref.)						
Married	−2.933	0.73	−0.256	−4.019	<0.001 ***	2.708
Divorced	−1.336	1.246	−0.054	−1.072	0.284	1.662
Gender						
Male (Ref.)						
Female	0.926	0.429	0.091	2.159	0.031 *	1.173
Residence						
City (Ref.)						
Country	−0.058	0.624	−0.004	−0.093	0.926	1.243
Rural	−1.783	0.787	−0.12	−2.265	0.024 *	1.866
Educational level						
College degree or above (Ref.)						
Senior high school or technical secondary school education	−0.092	0.789	−0.006	−0.117	0.907	1.873
Less than senior high school education	2.603	0.818	0.244	3.184	0.002 **	3.923
Religious						
No (Ref.)						
Yes	−0.856	1.096	−0.032	−0.78	0.436	1.149
Employment status						
Employed (Ref.)						
Retired	−0.873	0.835	−0.065	−1.045	0.296	2.609
Unemployed	0.788	0.719	0.057	1.095	0.274	1.807
Per capita monthly household income						
≤3000 CNY (Ref.)						
3001~5000 CNY	0.82	0.623	0.075	1.316	0.189	2.172
≥5001 CNY	2.447	0.664	0.239	3.685	<0.001 ***	2.801
Payment method of physical examination fee						
At one’s own expense (Ref.)						
The work unit pays	0.108	0.583	0.009	0.184	0.854	1.516
Health insurance card	2.423	0.492	0.231	4.927	<0.001 ***	1.469
Frequency of physical examination						
Annually (Ref.)						
Every 2–3 years	1.309	0.464	0.126	2.821	0.005 **	1.337
Every 4 years or more	3.132	0.645	0.248	4.858	<0.001 ***	1.731
Family genetic history						
No (Ref.)						
Yes	−0.971	0.992	−0.041	−0.979	0.328	1.164
Social support	0.259	0.028	0.417	9.321	<0.001 ***	1.332

*: *p* < 0.05, **: *p* < 0.01, ***: *p* < 0.001. Note: R = 0.638; R^2^ = 0.407; Adjusted R^2^ = 0.377; F = 13.552; *p* < 0.001. Abbreviations: B, unstandardized regression coefficients; β, standardized regression coefficients; SE, Std. error of B; Ref., Reference.

## Data Availability

The data presented in this study are openly available in Mendeley Data at doi:10.17632/vnh9t4ccxw.1.
